# Au@Pt Core-Shell Nanoparticle Bioconjugates for the Therapy of HER2+ Breast Cancer and Hepatocellular Carcinoma. Model Studies on the Applicability of ^193m^Pt and ^195m^Pt Radionuclides in Auger Electron Therapy

**DOI:** 10.3390/molecules26072051

**Published:** 2021-04-03

**Authors:** Kamil Wawrowicz, Agnieszka Majkowska-Pilip, Damian Gaweł, Ewelina Chajduk, Tadeusz Pieńkowski, Aleksander Bilewicz

**Affiliations:** 1 Centre of Radiochemistry and Nuclear Chemistry, Institute of Nuclear Chemistry and Technology, Dorodna 16 Str., 03-195 Warsaw, Poland; k.wawrowicz@ichtj.waw.pl (K.W.); a.bilewicz@ichtj.waw.pl (A.B.); 2 Department of Immunohematology, Centre of Postgraduate Medical Education, Marymoncka 99/103, 01-813 Warsaw, Poland; damian.gawel@cmkp.edu.pl; 3 Laboratory of Nuclear Analytical Techniques, Institute of Nuclear Chemistry and Technology, Dorodna 16 Str., 03-195 Warsaw, Poland; e.chajduk@ichtj.waw.pl; 4 Department of Oncology and Breast Diseases, Centre of Postgraduate Medical Education, Marymoncka 99/103, 01-813 Warsaw, Poland; tadeusz.pienkowski@cmkp.edu.pl

**Keywords:** core-shell nanoparticles, platinum, hepatocellular carcinoma, breast cancer, Auger electron therapy

## Abstract

^193m^Pt and ^195m^Pt radionuclides are therapeutically attractive Auger electron emitters with notably high Auger electron yield per decay. The present paper summarizes the first step of research on the applications of core-shell (Au@Pt) nanoparticles for electron Auger therapy of HER2+ (human epidermal growth factor receptor 2) breast cancer and hepatocellular carcinoma. Gold nanoparticles (30 nm) were synthesized covered with a platinum shell at high efficiency (>80%) and were further evaluated for in vitro studies such as binding affinity, internalization and cytotoxicity. To find the mechanism(s) responsible for platinum cytotoxicity in HepG2 cells, the platinum concentration in isolated cell nuclei and cytoplasm was determined using ICP-MS (inductively coupled plasma mass spectrometry). Lack of platinum in cell nuclei suggests that the cytotoxic effect is associated with the generation of reactive oxygen species (ROS) and reactive nitrogen species (RNS). Studies carried out on the SKOV-3 cell line with the use of a synthesized targeting bioconjugate (Au@Pt-PEG-trastuzumab) revealed a high affinity of this preparation to HER2+ cells, its internalization, its placement in the perinuclear area and partial intranuclear location. The specific binding for HER2 negative cells, MDA-MB-231, was negligible and Au@Pt-PEG-trastuzumab did not enter these cells. The results obtained are promising and warrant future investigation of Auger electron therapy using ^193m^Pt and ^195m^Pt based radiopharmaceuticals.

## 1. Introduction

In the last decade, there has been great emphasis on “targeted therapies” that are designed to kill cancer cells selectively, leaving healthy tissues unaffected. One such therapy is targeted radionuclide therapy (TRT) in which cancer cells are killed as a result of corpuscular radiation emitted by radionuclides conjugated to biological molecules that bind to the surface of specific cancer cells. The specificity of binding is affected by the recognition of surface receptors or other proteins overexpressed by cancer cells. Such a selective mechanism of radiation delivery minimizes toxicity to the surrounding normal tissues. Depending on the tumor size and location, the type of radiation selected is the critical factor for cancer treatment. In the case of small tumors and cancer metastasis treatment, remarkable therapeutic results have been achieved recently with the use of targeting molecules labeled with emitters of short distance corpuscular radiation like α and Auger electron particles [1,2].

The low-energy Auger electrons are emitted by radioisotopes that decay through the electron capture or internal conversion processes. Following this, vacancies in electronic shells are rapidly filled by electrons which drop in from external orbitals. Typically, an average of 5 to more than 35 Auger electrons, with energies ranging from a few eV to approximately 1 keV, are emitted per decaying atom. The linear energy transfer (LET) of Auger electrons ranges from 4 to 26 keV/µm [3]. Therefore, Auger electrons are similar to high-LET particles, like α particles, and produce highly damaging effects to cells. Because the path length of Auger electrons is short compared to the size of a cell, they are minimally toxic to surrounding non-targeted cells and they are therefore interesting candidates for targeted radionuclide therapy. However, the challenge is to target cancer cells specifically and achieve intracellular and intranuclear uptake for maximum DNA damage.

Among the many known Auger electron emitters, radionuclides ^193m^Pt and ^195m^Pt are therapeutically attractive due to very high Auger electron yield per decay. ^193m^Pt emits about 26 Auger electrons per decay and ^195m^Pt about 36 Auger electrons. The half-lives of both radionuclides, 4.33 and 4.03 days respectively, match the time of accumulation of monoclonal antibodies in cancer tissues. Interest in both platinum radionuclides is not only because of their suitable decay properties but also because platinum-complexes (like *cis*-platinum and carboplatinum) are commonly used in chemotherapy as anticancer drugs. Although the mechanism of action of these compounds at the molecular level is still unclear, it is widely accepted that Pt coordinates to N^7^ sites of DNA guanine bases to form adducts with both DNA helixes after hydrolysis of the Pt–Cl bonds [4]. This would block cell replication. While the high chemotoxicity of platinum coordination complexes is a limiting factor in their use in cancer therapy, the expected effects of radioplatinum with embedded high LET can be a unique approach to combine chemotherapy and radionuclide therapy. This “chemo-Auger” combination therapy may provide an opportunity to reduce chemotoxicity in critical organs while improving overall therapeutic efficacy [1,5]. Unfortunately, therapies similar to *cis*-platin have severe side effects because of low selectivity to cancer cells. Nonspecific targeting of ^193m,195m^Pt in complex compounds causes toxicity to normal tissues. Consequently, targeted delivery and controlled radioactive Pt ion release are essential to precise cancer therapy.

Contrary to the toxicity of *cis*-platin, which is activated simply in hydrophilic environment, metallic platinum is considered to be non-reactive because it can only dissolve in aggressive agents, such as aqua regia, which oxidizes it and then dissolves it [6]. On the other hand, taking into account the positive E^0^ (standard redox potential) value of the summary of reactions presented below, it can be assumed that in a strongly oxidizing environment and slightly acidic conditions, Pt^0^ can be spontaneously converted to Pt^2+^.

(1) $${\mathrm{Pt}}^{0}\;↔\;{\mathrm{Pt}}^{2+}\;+\;2e^{-}\;\;\;\;\;\;\;\;\;\;\;E^{0}\;=\;-1.18\;V$$

(2) $$H_{2}O_{2\;}+2H^{+}+2e^{-}\;↔\;2H_{2}O\;\;\;\;\;\;\;E^{0}=1.776\;V$$

(3) $${\mathrm{Pt}}^{0}\;+\;H_{2}O_{2}\;+\;2H^{+}\;↔\;{\mathrm{Pt}}^{2+}\;+\;2H_{2}O\;\;\;\;\;\;\;\Delta E\;=\;0.596\;V$$

The kinetics of this reaction are probably very slow and, in the macroscale, one could only observe minimal dissolution of Pt. However, it has recently been discovered that the degree of oxidation can be significantly increased by reducing the size of Pt to increase the surface to volume ratio, allowing oxygen adsorption and facilitating corrosive surface oxidation [6,7]. Chien et al. [8] and Shoshan et al. [9] reported that small non-radioactive Pt nanoparticles show very high cytotoxicity to the selected cancer cells with high oxidation potential. The authors explained the potent and selective cytotoxic effect of Pt nanoparticles by intracellular oxidation. This results in the loss of its intrinsic chemical inertness and a gain in its surface corrodibility for further dissolution in weakly acidic organelles, such as endosomes and lysosomes. This releases toxic Pt^2+^ ions that block cell division by binding to DNA and causing DNA damage. PtNPs should therefore have particularly high toxicity in cellular environments with a high oxidation potential. According to Szatrowski and Nathan [10] two aggressive types of cancer cells, hepatic (HepG2) and breast/ovarian HER2+ (SKOV-3), have a higher oxidative state than other cells owing to a high concentration of reactive oxygen species. Therefore, PtNPs should be ideally suited to target both of the aforementioned cancer cells [10]. Taking into account that in the case of Auger electron therapy, radionuclides must be located near to the cell nucleus so as to access the DNA strand (preferably intercalated). It can be seen that such conditions can be met by the ^193m,195m^Pt^2+^ cation released from radioactive ^193m,195m^PtNP in the cytoplasm with high oxidizing potential.

Another proposed mechanism of the cytotoxic action of platinum nanoparticles is mediated via the induction of generated reactive oxygen species (ROS) like ^●^O_2_ˉ, H_2_O_2_, ^●^OH, etc. and reactive nitrogen species (RNS), mainly ^●^NO [11,12]. The increased ROS and RNS cause membrane damage, mitochondrial dysfunction and oxidative DNA damage. In this case, replacement of non-radioactive platinum with Auger electron emitters ^193m^Pt or ^195m^Pt should greatly increase ROS and RNS generation and significantly increase cytotoxicity.

In this publication, the synthesis of platinum-coated gold nanoparticles, their conjugation to biomolecules and their physicochemical and biological characteristics are reported. In this research, instead of very small PtNPs (<2 nm), Au@Pt (30 nm) core-shell nanoparticles (NPs) were used, in which AuNPs were covered with a monolayer of Pt atoms. So, as in the case of very small Pt nanoclusters, all the Pt atoms are found on the surface of the nanoparticle. Presented studies are mainly focused on the preliminary experiments concerned on synthesis and characterization of functionalized Au@Pt core-shell nanoparticles as a promising tool for radiopharmaceuticals drug delivery. Taking into account the very short tissue range of Auger electron emitters, extensive in vitro studies were required to prescribe the intracellular penetration of synthesized conjugates. Platinum-based drugs mechanism of action is still questionable and unclear; thus, we present our findings related to this subject. Additionally, this research shows also multi-conceptual application of Au@Pt conjugates as targeted nanobrachytherapy approach, which may play a significant role in oncology as an extension of present therapeutic conventional methods.

Availability of platinum metastable radionuclides is strictly limited due to low reactor-produced radionuclides yield and requirement of high target material enrichment. Taking this into consideration, this paper may be meaningful and benefit in cancer therapy ground, specifically in nuclear medicine field. Thus, this work is the first in a series of publications where in novel radiotherapy alternative is under consideration.

## 2. Results and Discussion

### 2.1. Synthesis and Characterization of Au@Pt (30 nm)

**Figure 1:**
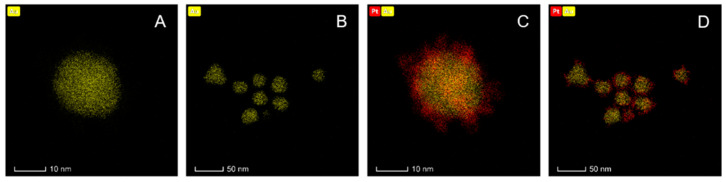
HR-TEM images for AuNP (**A**,**B**) and Au@PtNP (**C**,**D**).

**Figure 2:**
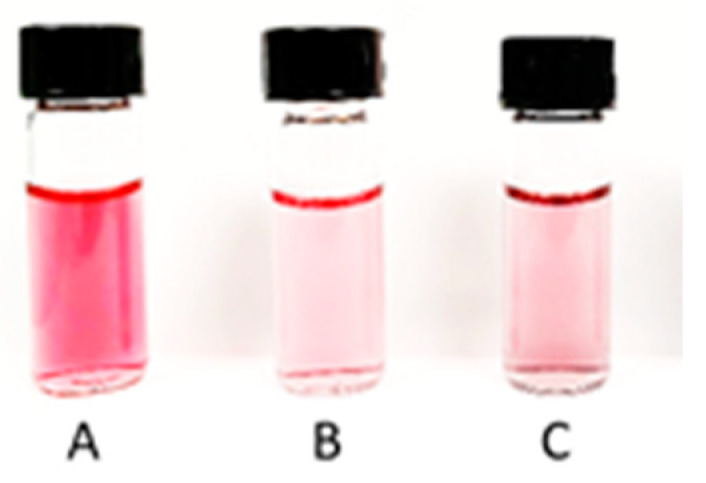
Digital image of synthesized AuNPs (**A**), Au@Pt–monolayer (**B**) and Au@Pt–multilayer (**C**).

**Figure 3:**
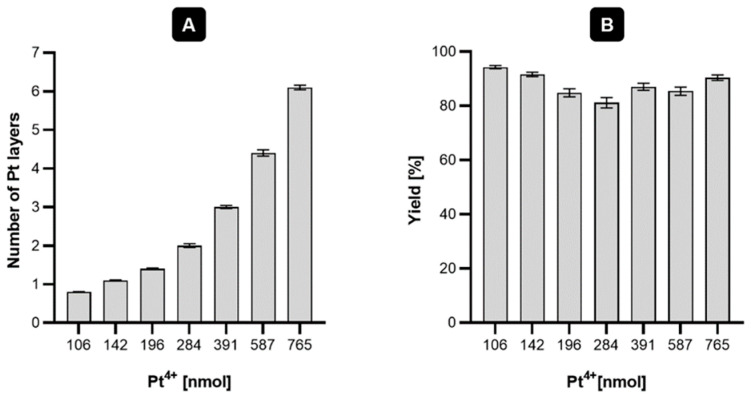
Graphical presentation of dependence the number of Pt layers formed (**A**) and yield of synthesis (**B**) in function of Pt^4+^ nmoles used for the synthesis.

In this study, it was decided to assess whether larger core-shell nanoparticles, such as platinum coated nanoparticles (Au@Pt) with the majority of Pt^0^ atoms also on the surface, exhibit similar properties. However, the most important goal of this work was to design radioactive Au@Pt labelled with two Auger emitters, ^193m^Pt and ^195m^Pt. For this study, gold AuNPs (30 nm) coated with one or more platinum layers were chosen. As mentioned by Gao et al. [13], to obtain ultra-thin platinum layers on the surface of gold nanoparticles it is necessary to find optimal amounts of Pt added to the reaction mixture. Synthesis of core-shell nanoparticles consists of slow, regular deposition and quick diffusion of reduced platinum atoms over the core area. Following this, platinum atoms may migrate onto the gold area and form a regular layer, filling up most of the AuNP surface. Images of HR-TEM (high-resolution transmission electron microscopy) presented in Figure 1 showed a relatively uniform deposition of platinum atoms on the AuNP surface. Figure 2 presents digital image of synthesized nanoparticles. Depending on the amount of platinum used for the synthesis, different numbers of monoatomic layers are formed on the AuNP surface (Figure 3). Assuming the uniform distribution of atoms on the AuNP surface, the Pt atoms monolayer should be obtained from 130 nmol of Pt^4+^ used for the synthesis. The obtained Au@PtNPs were characterized by DLS (Dynamic Light Scattering). As shown in Table 1, the hydrodynamic diameter of Au@Pt is slightly larger than the hydrodynamic diameter of the AuNP, which confirms the formation of Pt layers on the AuNPs surface. The large change in zeta potential is likely related to the different amounts of citrates adsorbed on the AuNP and Au@PtNP surface.

It must be noted that the synthesis of Au@Pt proceeds with a high yield (>80%), which is an important factor for further synthesis with radioactive materials. One critical aspect, in the context of the target synthesis with radioactive ^193m/195m^Pt, was to obtain an ultrathin (monoatomic) layer of platinum due to the very short range of Auger electrons emitted during platinum radionuclide decay. Additionally, this method of synthesis allows it to potentially induce biological toxicity due to increased catalytic activity of the platinum, which is much stronger for a thick platinum shell [14,15].

### 2.2. Synthesis of Au@Pt-PEG-COOH and Au@Pt-PEG-Trastuzumab Bioconjugate

**Figure 4:**
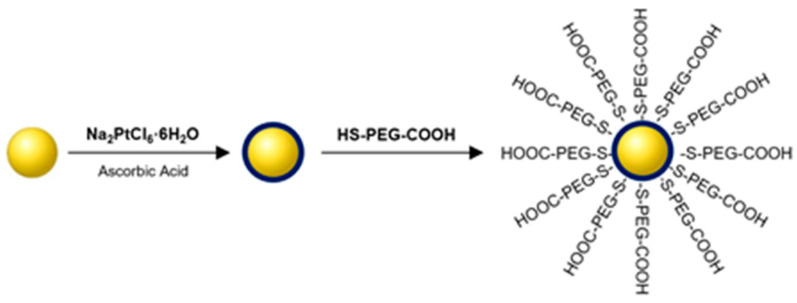
Schematic diagram of the synthesis of Au@Pt-PEG-COOH conjugate.

Synthesis of Au@Pt-PEG-COOH (Figure 4) was performed in a one-step process. To improve the dispersity and stability of NPs, 15,000 molar excess of HS-PEG-COOH (thiolated carboxylic-polyethylene 4α;-sulfanyl—ω carboxy PEG) ligand was used. After the synthesis, unreacted PEG was removed by centrifugation and the NPs were dispersed in water. The obtained product was characterized by DLS (dynamic light scattering) and successful conjugation of PEG chains to nanoparticles was confirmed by a significant increase of size and zeta potential (Table 2).

**Figure 5:**
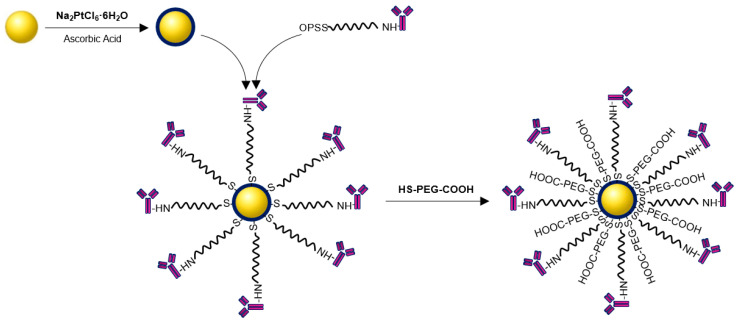
Schematic diagram of the synthesis of Au@Pt-PEG-trastuzumab bioconjugate.

Synthesis of the Au@Pt-PEG-trastuzumab bioconjugate was performed as reported previously [16]. The first step relied on the conjugation of trastuzumab with orthopyridyldisulfide-polyethyleneglycol-succinimidyl carboxymethyl ester (OPSS-PEG-NHS) to form a stable peptide bond. Further, after removing unreacted PEG, OPSS-PEG-trastuzumab was coupled with core-shell Au@Pt nanoparticles. Additionally, to increase the stability and dispersity of the bioconjugate, PEGylation with HS-PEG-COOH (5 kDa) was performed. The scheme of the bioconjugation is shown in Figure 5.

Successfully obtained bioconjugate was characterized using DLS by measuring the hydrodynamic diameter and zeta potential. The significant change of these parameters after each step of the synthesis confirms efficient conjugation of OPSS-PEG-trastuzumab (I step) and HS-PEG-COOH (II step). Zeta potential > −30 mV suggested high stability of the synthesized bioconjugate, which was confirmed in other studies in different media conditions.

Application of the radiometric method with ^131^I labeled trastuzumab allowed for the determination of the number of trastuzumab molecules attached to one Au@PtNP. The calculations revealed that bioconjugates contained an average of 22.4 ± 1.4 trastuzumab molecules per nanoparticle.

**Figure 6:**
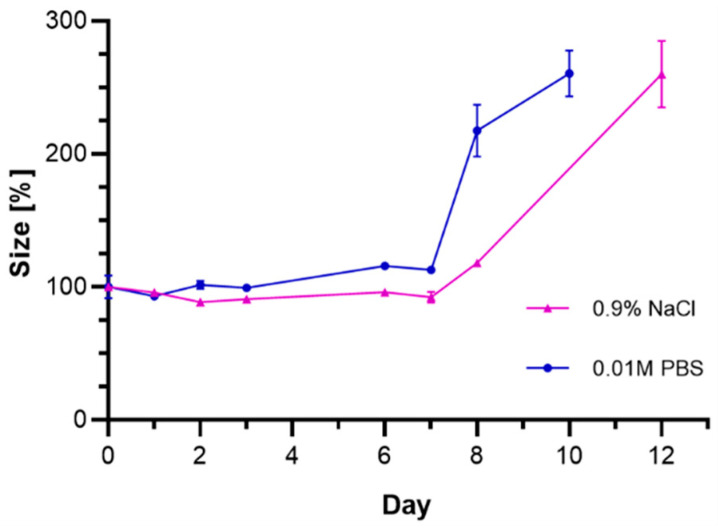
Changes in the hydrodynamic diameter of the Au@Pt-PEG-trastuzumab bioconjugate incubated in 0.9% NaCl and 10 mM phosphate-buffered saline (PBS) buffer at 37 °C.

To characterize the rate of chemical degradation of the synthesized bioconjugates, stability studies in PBS buffer (pH = 7.4) and saline solutions were carried out (Figure 6). Unfortunately, using several different types of media, including biological fluids, performing such experiments in human serum (HS) or in growing medium containing fetal bovine serum (FBS) was unsuccessful due to the presence of large amounts of proteins, which alter the DLS measurement results.

The stability of the bioconjugate was determined by DLS through the measurement of hydrodynamic diameter over time. The tested compound started to lose stability after 7 days from the beginning of the experiment which might suggest the agglomeration of the bioconjugate. Considering the future use of bioconjugates labeled with ^193m^Pt (t_1/2_ = 4.33 d) and ^195m^Pt (t_1/2_ = 4.03 d) radionuclides, the obtained results indicate sufficient stability to perform targeted therapy.

### 2.3. In Vitro Experiments

**Figure 7:**
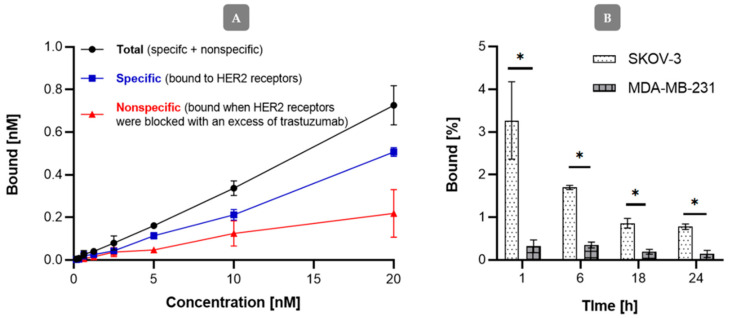
Binding studies of Au@Pt-PEG-[^131^I]trastuzumab on SKOV-3 cell line (**A**) and comparison of binding Au@Pt-PEG-[^131^I]trastuzumab on SKOV-3 (HER2+) and MDA-MB-231 (HER2−) cell lines (**B**). For statistical analysis, *p* values were calculated using Student’s *t*-test (* *p* < 0.05).

The affinity of the radiobioconjugate to HER2 receptors was examined on SKOV-3 (HER2+) and HER2 negative MDA-MB-231 cell lines with the use of iodinated Au@Pt-PEG-[^131^I]trastuzumab applying the same procedure as for other nanoparticle-trastuzumab bioconjugates [17,18]. Figure 7A shows significant differences of binding in the presence and in the absence of free trastuzumab used in 100-fold molar excess to block the receptors. The results indicate that the Au@Pt-PEG-[^131^I]trastuzumab bioconjugate binds specifically to the HER2 receptor on SKOV-3 cells. As presented in Figure 7B, in the case of the HER2 negative MDA-MB-231 cell line, the specific binding is negligible.

**Figure 8:**
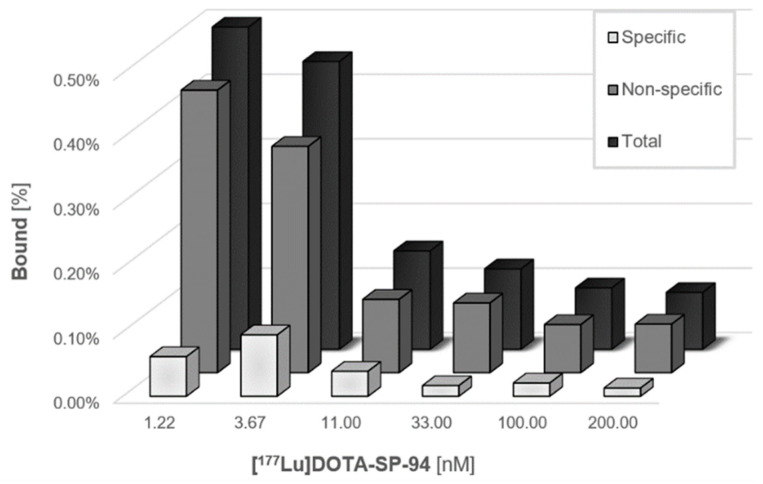
Graphical presentation of [^177^Lu]DOTA-SP-94 binding to HepG2 cells.

Unfortunately, we did not obtain satisfactory results of binding studies of Au@PtNP-SP-94 conjugate to glucose regulated protein (GRP78) receptors present on HepG2 cells. Despite the existence of a literature report [19], where the authors found significant receptor affinity of the ^99m^Tc-HYNIC-SP-94 radiobioconjugates for GRP78 receptors [20] on HepG2 cells, in the case of [^177^Lu]DOTA-SP-94 only very low specific binding of the SP-94 peptide was observed (Figure 8). This indicates a very small number of GRP78 receptors on HepG2 cells or a low affinity of the biomolecule to this cell membrane receptor. The highest binding percentage was found for two of the lowest doses (1.22 nM and 3.67 nM). The very small receptor affinity does not allow the use of our proposed radioactive Au@^193,195m^PtNP-SP-94 for targeted therapy and restricts the use of nanoparticles only for local therapy, namely, nanobrachytherapy. Therefore, in the case of Au@PtNP, studies were limited to internalization and cytotoxicity tests of the Au@PtNP-PEG-COOH conjugate.

**Figure 9:**
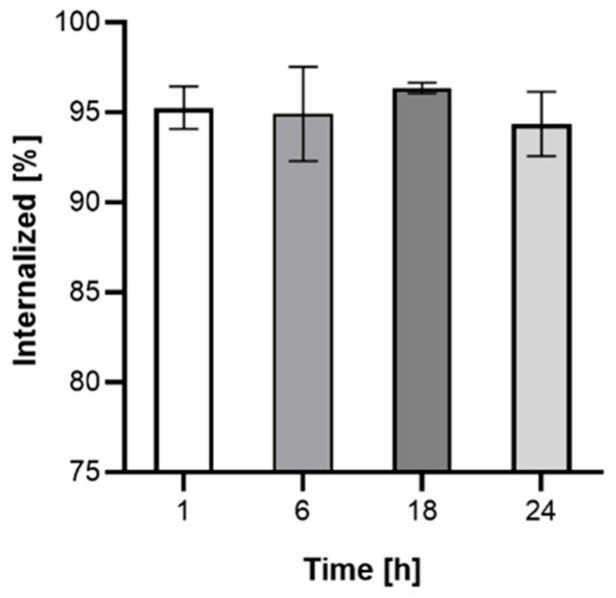
Internalization kinetics of Au@Pt-PEG-[^131^I]trastuzumab on SKOV-3 cells.

Due to the very short range of Auger electrons, internalization of the radiobioconjugate is required to achieve a therapeutic effect. The kinetics of internalization were performed on the SKOV-3 cell line with the use of Au@Pt-PEG-[^131^I]trastuzumab. As presented in Figure 9, more than 90% of bioconjugate internalizes after 1 h and remains at a similar level for up to 24 h. Due to the lack of specific binding, internalization studies were not conducted on MDA-MB-231 cells.

**Figure 10:**
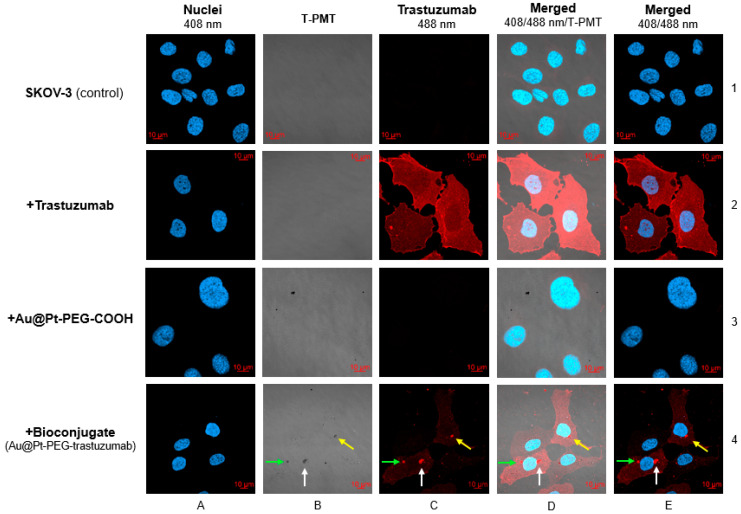
Confocal images of SKOV-3 cells treated with trastuzumab, Au@Pt-PEG-COOH and Au@Pt-PEG-trastuzumab conjugates. As a control untreated cells were used. Blue fluorescence signal–DAPI staining of cell nucleus; red fluorescence signal–trastuzumab accumulation; black spots–nanoparticles visualized using transmitted light detector (T-PMT). Corresponding signals are marked with colored arrows.

To confirm the capability of SKOV-3 cells to internalize the synthesized bioconjugates, cells were exposed to trastuzumab, Au@Pt-PEG-COOH and Au@Pt-PEG-trastuzumab for 24 h. Figure 10 summarizes the confocal microscopy imaging experiment. As expected, only bioconjugate Au@Pt-PEG-Trastuzumab was effectively internalized and localized into SKOV-3 cells cytoplasm. Presented in panel B/4 dark spots reflect Au@PtNPs, while red fluorescence signal is related to the trastuzumab bound (panel C/4). For unambiguous intracellular uptake evaluation, cells nuclei were stained with blue-fluorescent signaling DAPI (2-(4-Amidinophenyl)-6-indolecarbamidine dihydrochloride, 4′,6-Diamidino-2-phenylindole)). Merged signals presented in panel D/4 and E/4 disclosed the successful bioconjugate penetration of SKOV-3 cells and its localization in the perinuclear area. As expected, non-targeted Au@Pt-PEG-COOH was not internalized; thus, no signal inside the cells was detected (panel B/3).

**Figure 11:**
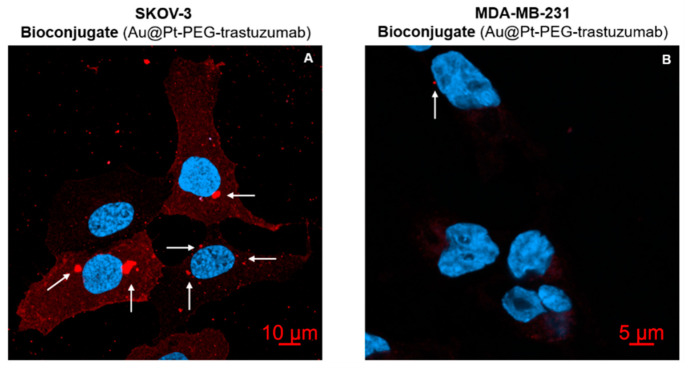
Merged 408/488 nm images for bioconjugate treated SKOV-3 and MDA-MB-231 cell line. Intensified red signal corresponds to bioconjugate accumulation (marked with white arrow).

Figure 11 shows a comparison of merged 408/488 nm signals for SKOV-3 and MDA-MB-231 cells. The presented images show that Au@Pt-PEG-trastuzumab bioconjugate can be internalized only into HER2 overexpressed SKOV-3 cells, whereas they do not enter cells without HER2 overexpression.

**Figure 12:**
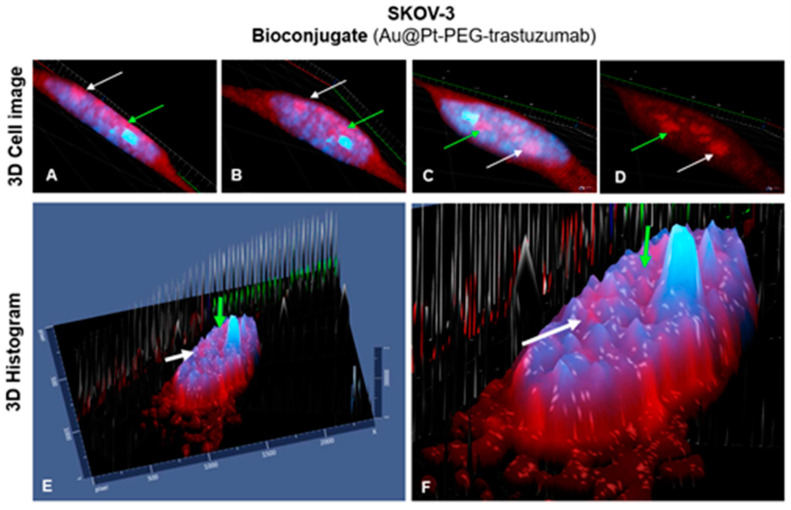
3D confocal images of SKOV-3 cell treated with Au@Pt-PEG-trastuzumab bioconjugate. Corresponding signals are marked with green and white arrow in each picture, respectively. (**A**–**D**) 3D images of differently rotated cell; (**E**,**F**) 3D histograms.

Interestingly, these results strongly indicate that synthesized bioconjugate Au@Pt-PEG-trastuzumab can internalize not only into the cell but even into the cell nucleus. As shown in Figure 12, several individual signals which are presented on the nuclei area may prove that some single parts of the applied bioconjugate were internalized into the cell nucleus. This may be particularly important in the treatment performed with radiobioconjugates and may affect an increase in cytotoxicity. In Figure 12A–D are marked 3D images of SKOV-3 single cell, while E and F are 3D histograms showing that the presence of intensified red signals on the nucleus area is an effect of the internalized bioconjugate rather than the bioconjugate localized around the nucleus. It needs to be highlighted that the absence of black signal (T-PMT) of nanoparticles is an aftermath of creating 3D images and it is impossible to observe the three-dimensional signal of the transmitted-light detector.

Despite the impossibility to perform experiments on HepG2 cells with the use of the targeting conjugate, the internalization of PEGylated core-shell nanoparticles via passive transport was examined. According to the literature data, passive transport of nanoparticles is highly dependent on several factors including shape, surface charge, size and hydrophobicity. Moreover, the characteristics of the cancer cell are also key parameters that play a crucial role in passive transport of different types of nanomaterials and their further metabolization inside cell plasma [21]. Several models determine the “optimal” conditions for passive internalization of nanomaterials. Most of them presume that during this process cell membrane deformation is achieved and defines ~50 nm diameter of nanoparticles as optimal [22].

**Figure 13:**
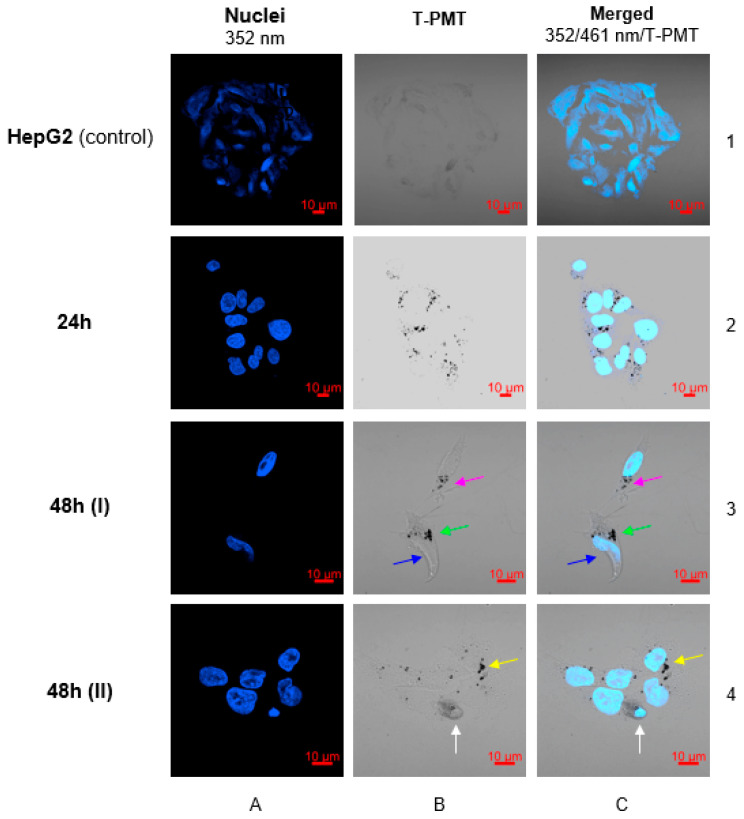
Confocal microscopy images for HepG2 cell line treated with Au@Pt-PEG-COOH after 24 and 48 h. Blue fluorescence—cell nuclei visualized with Hoechst 33258; dark spots—nanoparticles visualized by T-PMT detector. Corresponding signals of nanoparticles in cell area were marked with colored arrows.

Confocal microscopy images (Figure 13) clearly show successful internalization of the conjugate into the cell. Since receptor-mediated endocytosis is a faster process than passive transport, it was decided to perform imaging after 24 and 48 h. As presented in Figure 13 (panels B/2 and C/2), 24 h is sufficient to localize the conjugate inside the cell and around the cell nucleus. In images performed after 48 h, the first symptoms of cytotoxicity were observed. In panel C/3 a contrail of Hoechst signal was observed, which is characteristic to the disintegration of the cell nucleus as a result of the action of toxic conjugate localized around the nuclear envelope (green arrow). Moreover, the forming of apoptotic bodies (blue arrow) which contain concentrated cytoplasm, chromatins and cellular organelles covered by cell membrane was observed. This is typical for programmed cell death–apoptosis (Figure 13, panel C/3). In the second image performed after 48 h (Figure 12, panel C/4), an intensified blue signal from the nucleus was observed. This is an additional confirmation of the cytotoxic mechanism of action of the core-shell nanoparticles conjugate, which effects with enhanced permeability of cell and nucleus membrane for Hoechst dye (white arrow). Due to the inability of using any stainer in visualized conjugate, it was not possible to perform 3D images.

**Figure 14:**
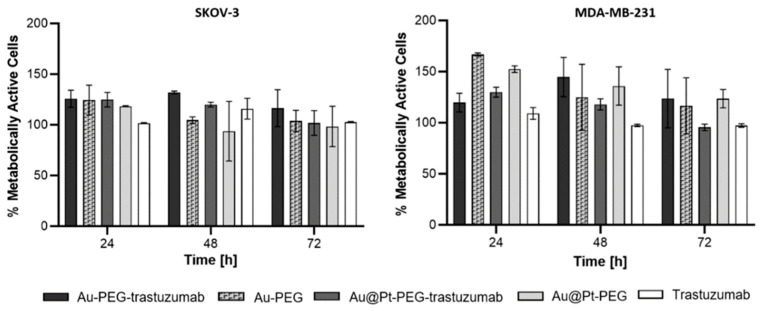
Metabolic activity of SKOV-3 and MDA-MB-231 cell after exposure for AuNP and Au@Pt conjugates.

**Figure 15:**
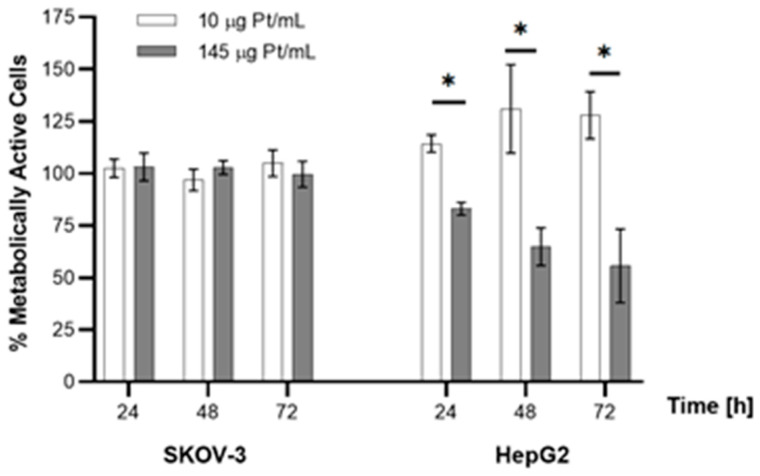
Dependence of metabolic activity of SKOV-3 and HepG2 cells on contents of Pt on Au@PtNP nanoparticles. For statistical analysis, *p* values were calculated using one-way analysis of variance (one-way ANOVA) (* *p* < 0.05).

For confocal microscopy, the concentration of nanoparticles was referenced against the dose of 5 nM of bioconjugate used in SKOV-3 imaging. Cytotoxicity experiments with the use of the MTS assay (Figure 14 and Figure 15) showed that this concentration did not induce cytotoxicity of HepG2 cells. As reported previously [9], Pt^0^ atoms are nontoxic, whereas oxidation into Pt^2+^ may cause cytotoxicity. To achieve Pt^0^ dissolution, a highly oxidative environment inside cells is required. Based on literature data, SKOV-3 cells have slightly increased hydrogen peroxide levels [10] which have drawn attention to this type of cell line. Figure 14 represents the results of cytotoxicity studies performed on SKOV-3 and MDA-MB-231 cells with the use of Au@Pt-PEG and Au@Pt-PEG-trastuzumab conjugates. As a control, AuNP conjugates not covered with platinum layers were used. Toxicity was not observed in any of the tested systems. It is worth noting that free trastuzumab in concentrations used in the bioconjugate did not induce any cytotoxicity which is an effect of the low content of antibody linked to the conjugate. In the synthesized compound, trastuzumab’s function is limited to acting as a targeting molecule, without any expectations of inducing toxicity.

The lack of cytotoxicity is probably related to the low concentration of platinum atoms in the Au@Pt-PEG-trastuzumab bioconjugate and not enough ROS concentration in the cells tested. In the publications where the cytotoxic mechanism of PtNPs related to the dissolution of Pt atoms has been described [6,7] authors tested cell lines with a significantly higher level of H_2_O_2_ production, e.g., lung cancer cell lines or hepatocellular carcinoma. Another important factor is associated with the concentration of platinum ions. In studies describing the toxicity of Pt^2+^ cations released from PtNPs, much higher concentrations of platinum were used in comparison to the experiments presented in this work [12]. Based on these data, studies were performed comparing the cytotoxic effect of AuNP covered by a monolayer of Pt atoms (10 µg Pt/mL) and AuNP covered by few layers of Pt atoms (145 µg Pt/mL). The experiments were performed on SKOV-3 and HepG2 cells treated with Au@Pt-PEG-trastuzumab and Au@Pt-PEG-COOH, respectively. As presented in Figure 15, no toxicity was observed for the SKOV-3 cell line in both Pt concentrations. However, the results were quite different for HepG2 liver cancer cells where multilayer Au@PtNPs induced toxicity with a gradual decrease of metabolic activity of cells after 24, 48 and 72 h.

Based on the positive confocal imaging results for the Au@Pt conjugates, the ability of this conjugate to overcome the nuclear envelope and go through the cytoplasm into the nucleus was examined. As mentioned previously, the transport of Pt^2+^ ions to the cell nucleus has been postulated many times as a source of the cytotoxic effects of very small (~2 nm) PtNPs [9,23]. However, concentrations of Pt in the cell nucleus have not been measured so far. The nuclei isolation process was performed with a high yield of 94.8 ± 8.2%, which is in accordance with the manufacturer’s instructions for adherent cells. Determination of platinum by ICP-MS of two separate fractions, cytoplasm and nucleus, proves that platinum is localized in the cytoplasm after internalization. Unfortunately, significant Pt transport to the cell nucleus was not noted. The ultra-trace level of Pt observed in the nucleus (~0.5 fg Pt/nucleus) was probably derived from Pt contamination of the nucleus fraction during the separation process (Table 3). The obtained results are in contrast, to the results obtained by Shoshan et al. [9], where for 2 nm PtNPs HepG2 cells, about 70% of the Pt had accumulated in HepG2 cell nuclei.

Additionally, the platinum to gold mass ratio is at a similar level compared to the value in the synthesized compound (1.13–1.24 in cells vs. 1.07 in the synthesized compound), which indicates the lack of dissolution of atoms in the cytoplasm and their transport to the nucleus.

Hence, it is possible that the mechanism of Pt transport to the nucleus does not work in the case of larger core-shell Au@PtNPs or the postulated mechanism of Pt^2+^ transport to the nucleus is not correct.

Biological activity of platinum-based drugs is still under investigation and there is no consistent and experimentally confirmed mechanism of cytotoxic effect for complex compounds (i.e., *cis*-platin) or metallic platinum in form of PtNPs or core-shell nanoparticles.

Several published research works present different views on platinum nanoparticles cytotoxicity. Pedone et al. [24] reported that PtNPs do not induce cytotoxicity, whereas other sources clearly prove therapeutic anticancer activity of these compounds [9,25]. PtNPs ability to scavenging of superoxide radical (^●^O_2_^−^), hydrogen peroxide (H_2_O_2_) and hydroxyl radical (^●^OH) along with increase the level of the most important antioxidants, including glutathione (GSH), superoxide dismutase and malondialdehyde may affect in protecting the cells from oxidative stress even more effective than *cis*-platin. Additionally, as was reported, cytotoxic activity of PtNPs in HepG2 cells is caused by oxidation Pt^0^ to Pt^2+^ and further cell-cycle arrest and DNA damage via intercalation [11,25,26]. Thus, the mechanism of PtNPs cytotoxicity against HepG2 requires additional studies.

As mentioned, our studies are dedicated to application of core-shell Au@PtNPs labeled with ^193m^Pt and ^195m^Pt for Auger electron therapy. According to formerly proposed mechanism of PtNPs cytotoxicity through platinum ion intercalation into the DNA, therapeutic efficacy might be an effect of double-strand brakes formed after Auger electrons emission. Unfortunately, in our studies, we did not confirm platinum presence inside the HepG2 cell nucleus. However, effectiveness of treatment should be achievable using radioactive Au@^193m,195m^PtNPs and alternative “targets” in cell [27]. Due to intracellular localization of our Au@Pt-PEG-COOH and Au@Pt-PEG-trastuzumab conjugates we predict that therapy efficacy will be achievable by high radiation generation of ROS, which will diffuse freely into the intranuclear space and localize nearly to DNA. Additionally, we suppose, that emitted Auger electrons and free radicals formed after neutralization of highly charged-Pt^26+^ and Pt^36+^-cations may disintegrate the nuclear envelope. It must be noted that our conjugates not only internalize into the cytoplasm, but also locates in the perinuclear area what may be a crucial parameter when ^193m,195m^Pt will be used. During anaphase of mitosis, chromosomes are separated into two chromatids and located on the opposite sides of cell. This makes the genetic material in range of internalized Auger electrons and may also improve treatment efficacy. Emission of Auger electrons in the cytoplasm is associated with many physiological and morphological changes in a single cell. This includes disruption of electrons transport into the mitochondria and decrease of ATP level after cell membrane damage. Furthermore, passive transport of sodium and calcium cations into the cell affects in activation the nucleases and other DNA-cutting enzymes. As a result of this, disintegration of cell nucleus is observed and cell death is achieved [28].

## 3. Materials and Methods

### 3.1. Chemical Reagents

The chemical reagents used were as follows: gold (III) chloride trihydrate (HAuCl_4_·3H_2_O), trisodium citrate dihydrate (C_6_H_9_Na_3_O_9_), sodium hydroxide (NaOH), sodium hexachloroplatinate hexahydrate (Na_2_PtCl_6_·6H_2_O), ascorbic acid (C_6_H_8_O_6_), HS-PEG-COOH (5 kDa), paraformaldehyde (PFA), were purchased form Millipore Sigma (St. Louis, MO, USA). Orthopyridyldisulfide-polyethyleneglycol-succinimidyl carboxymethyl ester (OPSS-PEG-NHS, 5 kDa) was purchased from Creative PEGworks (Chapel Hill, NC, USA), iodogen (1,3,4,6-tetrachloro-3R,6R-diphenylglycouril) was obtained from Thermo Fischer Scientific (Waltham, MA, USA). Trastuzumab was isolated form Herceptin^®^ (Roche Pharmaceuticals, Basel, Switzerland). Biological experiments were performed with using following materials: growing medias-McCoy’s 5A, Dulbecco’s modified eagle medium (DMEM), Eagle’s minimal essential medium (EMEM); Trypsin EDTA solution C, phosphate-buffered saline (PBS) and fetal calf serum from Biological Industries (Beith Haemek, Israel), dimethylsulfoxide (DMSO) and the CellTiter96^®^ AQueous One Solution Reagent (MTS compound) from Promega (Mannheim, Germany). SKOV-3, MDA-MB-231 and HepG2 cells were obtained from the American Type Tissue Culture Collection (ATCC, Rockville, MD, USA) and cultured accordingly to the ATCC protocol. SP-94 and DOTA-SP-94 peptides were synthesized by NOVAZYM (Poznan, Poland) and CASLO ApS, c/o Technical University of Denmark (Kongens Lyngby, Denmark), respectively. For staining nucleus in SKOV-3 and MDA-MB-231 cell lines, DAPI purchased from Millipore Sigma (St. Louis, MO, USA) was used, whereas for staining HepG2 nucleus Hoechst 33258 (Ph–enol, 4-[5-(4-methyl-1-piperazinyl)[2,5’-bi-1H-benzimidazol]-2’-yl]-,trihydrochloride) purchased from Thermo Fischer Scientific (Waltham, MA, USA) was used. As mounting solution for confocal microscopy samples preparation DAKO Fluorescent Mounting Medium (Agilent Technologies, Santa Clara, CA, USA) was employed.

### 3.2. Radionuclides

For radioiodination of trastuzumab ^131^I radionuclide was applied. Na^131^I (no-carrier-added, with the specific activity of 550 GBq/mg) was obtained from POLATOM Radioisotope Centre (Świerk, Poland). Labelling of DOTA-SP-94 peptide was performed with ^177^Lu in the form of ^177^LuCl_3_ (specific activity of >370 GBq/mg) obtained from POLATOM Radioisotope Centre (Świerk, Poland).

### 3.3. Characterization Techniques for Nanoparticles

Size and zeta potential of nanoparticles were characterized with dynamic light scattering technique (DLS, Malvern, UK) and ultraviolet-visible spectroscopy (UV-Vis) (Thermo Fischer Scientific-Waltham, MA, USA). Size, shape and platinum layer collocation were determined by HR-TEM Microcopy (TALOS™ F200X, Thermo Fischer Scientific-Waltham, MA, USA) and ICP-MS spectrometer (Elan DRC II, Perkin Elmer, Waltham, MA, USA). Radioactivity of samples with [^131^I]trastuzumab and [^177^Lu]DOTA-SP-94 was determined with Wizard^2^ Gamma Counter (Perkin Elmer, Waltham, MA, USA). Stability control of [^131^I]trastuzumab during the synthesis was achieved by Thin Layer Chromatography technique with the use of Storage Phosphor System Cyclone Plus (Perkin Elmer, Waltham, MA, USA), glass microfiber chromatography paper impregnated with silica gel (iTLC SG, Agilent Technologies, Santa Clara, CA, USA) and with PBS buffer as mobile phase. All solutions were prepared with using ultrapure deionized water (18.2 MΩ·cm, Hydrolab, Straszyn, Poland).

### 3.4. Synthesis and Characterization of Au@Pt (30 nm)

AuNPs were synthesized in aqueous solution by citrate reduction method as described [29]. Briefly, 4.95 mg of HAuCl_4_·3H_2_O was alkalized with NaOH to pH~4.5 and heated 30 min in 95 °C. Afterwards, sodium citrate dihydrate as reductor was added (340 mM, 170 μL) and heating was continued for 3 h with color-change from yellow to red. Subsequently, the reaction mixture was cooled to room temperature and size and zeta potential were measured. Concentration of AuNPs was estimated from the mass of gold used to the synthesis and assuming the spherical shape of 30 nm nanoparticle. In the next step, AuNPs were covered with ultrathin layer of platinum via chemical reduction of Na_2_PtCl_6_·6H_2_O by ascorbic acid (AA) [13]. Initially, AuNPs solution was heated to 90 °C for 10 min. Afterwards, sodium hexachloroplatinate (0.32 mg, 1 mM) and ascorbic acid (28 mg, 4 mM) were step-wise added at 10 min and 30 min intervals. Subsequently, the reaction mixture was heated 30 min in 90 °C, cooled to room temperature and, finally, characterized by DLS and UV-Vis techniques.

Size and shape of the synthesized nanoparticles were measured using HR-TEM microscopy. The number of platinum atoms on AuNPs surface was determined by ICP-MS spectrometry. Calculation of the platinum layer thickness was performed as follows: 1 mL of supernatant after Au@Pt centrifuging was measured using ICP-MS what allowed to estimate the yield of the synthesis. The number of Pt-atoms on AuNP was calculated by dividing the conjugated quantity of Pt-atoms by the number of AuNPs. The number of monatomic layers on the surface was determined assuming a Pt atom diameter of 136 pm.

### 3.5. Synthesis of Au@Pt-PEG-COOH and Au@Pt-PEG-Trastuzumab Bioconjugate

PEGylated core-shell nanoparticles were synthesized by adding 15,000 molar excess of HS-PEG-COOH and mixing for 15 min, 30 min, 45 min, 1 h, 6 h, 12 h and 24 h to estimate the minimal time period for successful stabilization. For further experiments, including in vitro studies, 30 min PEGylation duration has been chosen as optimal.

Bioconjugate was synthesized as described by Cai et al. [16]. Trastuzumab (200 μg) was reacted with 25-molar excess of OPSS-PEG-NHS (5 kDa) in carbonate buffer (100 mM) overnight. Unreacted OPSS-PEG-NHS was removed during the purification of OPSS-PEG-trastuzumab with the use of centrifugal concentrators Vivaspin^®^500 100 kDa cut-off (Sartorius, Goettingen, Germany). Further, 7 μg of OPSS-PEG-trastuzmuab was reacted with 3 mL of previously purified and concentrated Au@Pt in 20 mM carbonate buffer for 45 min in Protein LoBind Tubes (Eppendorf, Hamburg, Germany). In the next step, to remove all unconjugated protein, Au@Pt-PEG-trastuzumab was centrifuged (6500 rpm) and dispersed in 20 mM carbonate buffer. Additionally, to increase the dispersity of bioconjugate, 15,000-molar excess of HS-PEG-COOH (5 kDa) was added. The final product was purified by centrifuging (6500 rpm), dispersed in ultrapure deionized water and characterized by DLS technique.

### 3.6. Determination of Trastuzumab Molecules Conjugated to Au@Pt Nanoparticles

For calculation of protein molecules conjugated to nanoparticles it was required to use iodinated trastuzumab ([^131^I]trastuzumab) applying Iodogen method [30]. Briefly, 1.5 mg of trastuzumab, 30 MBq of ^131^I and PBS buffer were mixed with Iodogen reagent for 10 min in room temperature. Purification of mixture was performed on PD-10 column (Sephadex G-25 resin; Millipore Sigma, St. Louis, MO, USA) and 10 mM PBS buffer as a mobile phase was used. Buffer exchange was carried out by using Vivaspin^®^500 centrifugal concentrators and the product was transferred into aqueous solution. Afterwards, 200 μg of [^131^I]trastuzumab was mixed with 25-fold molar excess of OPSS-PEG-NHS (5 kDa) in carbonate buffer (100 mM) overnight, purified and conjugated to nanoparticles. To calculate the yield of conjugation, supernatant after centrifuging of nanoparticles was collected and the activity of both-nanoparticles and supernatant-fractions was measured. Finally, determination of the number of iodinated trastuzumab’s attached to Au@Pt was calculated by dividing the moles of protein by the moles of nanoparticles.

### 3.7. Stability Studies of Au@Pt-PEG-Trastuzumab Bioconjugate

One mL of Au@Pt-PEG-trastuzumab was centrifuged and dispersed in PBS buffer and saline to determine the stability of bioconjugate in physiological conditions. Experiments were performed in 37 °C for 12 days (PBS and saline). Aggregation tendency was estimated by the hydrodynamic diameter and zeta potential measurements by DLS.

### 3.8. Receptor Binding Studies

Receptor binding affinity of synthesized Au@Pt-PEG-[^131^I]trastuzumab bioconjugate was performed on SKOV-3 cells overexpressing HER2 receptors cultured in McCoy’s 5A Modified medium supplemented with 10% of fetal bovine serum and 1% of penicillin-streptomycin. The day before the experiment, cells (6.0 × 10^5^) were seeded into six-well plates and incubated in 37 °C with 5% CO_2_ atmosphere overnight. Subsequently, cells were washed once with PBS. Next, one mL of various concentrations of bioconjugate was added and 1.5 h incubation was performed. Further, the medium was aspirated and cells were rinsed with PBS twice. In the last step, the cells were lysed thrice with 1 M NaOH and all of the fractions were measured by Wizard^2^ Detector Gamma Counter. For evaluation of nonspecific binding, HER2 receptors were blocked with 100-molar excess of non-conjugated trastuzumab. To calculate the specific binding, difference between total and nonspecific binding was quantified. Presented results (mean with SD) contain the data from two individual experiments, wherein each sample was repeated twice.

Binding affinity studies of the DOTA-SP-94 conjugate were performed as follows-15 nmol of DOTA-SP-94 peptide was labeled with 10 MBq of ^177^Lu for 30 min in 95 °C. After overnight incubation of 6.0 × 10^5^ cells in six-well plates and washing the cells with PBS, medium was replaced with various concentrations of [^177^Lu]DOTA-SP-94 ranging from 1.2 nM to 200 nM and incubated for 1.5 h. The medium was removed and cells were washed with PBS. In the last step, after medium removing and washing with PBS, the cells were lysed thrice with 1 M NaOH and all of the fractions were measured on a Wizard^2^ Detector Gamma Counter. Nonspecific binding was assessed by receptor blocking with 1000-fold molar excess of DOTA-SP-94 or SP-94 peptides. Specific binding was calculated as the difference between total and nonspecific binding. Results are presented as the mean with SD of three individual experiments, with two repeats of each sample.

### 3.9. Internalization Studies

Internalization studies were performed on SKOV-3 and MDA-MB-231 cells (McCoy’s 5A Modified and DMEM with 10% FBS and 1% penicillin-streptomycin supplementation respectively). Briefly, 6.0 × 10^5^ cells were seeded into 6-well plates and incubated 24 h in 37 °C with 5% CO_2_ atmosphere. After incubation, medium was replaced with 5 nM of bioconjugate in 1 mL of medium and incubated 1 h in 4 °C to avoid internalization. Subsequently, doses were collected into 4 mL plastic vials, cells were washed with PBS and 1 mL of fresh medium was added. Plates were incubated in four different time periods–1 h, 6 h, 18 h and 24 h. Consequently, medium was removed and cells were washed with PBS. To distinguish between membrane-bound (acid releasable) and internalized (acid resistant) radiocompound, the cells were washed twice with a 0.05 M glycine-buffer (pH 2.8) on ice. Afterwards, cells were lysed with 1 M NaOH and collected as internalized fraction. All fractions were measured with Wizard^2^ Detector Gamma Counter. Nonspecific binding was evaluated by blocking of HER2 receptors with 100-molar excess of non-conjugated trastuzumab. To calculate the specific binding, difference between total and nonspecific binding was quantified. Presented results (mean with standard deviation [SD]) contains the data from two individual experiments, with three repeats of each sample.

### 3.10. Cytotoxicity Studies

Cytotoxicity studies were performed on SKOV-3, MDA-MB-231 and HepG2 (cultured in EMEM with 10% FBS and 1% penicillin-streptomycin supplementation) cells with the MTS assay. Briefly, 3 × 10^3^ cells were seeded into 96-well plates and incubated 24 h in 37 °C with 5% CO_2_ atmosphere. After incubation, the medium was removed and cells were washed with PBS. Subsequently, Au@Pt-PEG-trastuzumab bioconjugate (SKOV-3 and MDA-MB-231) or Au@Pt-PEG-COOH (HepG2) was added and incubated 24 h, 48 h and 72 h. Before the incubation with MTS reagent, medium was removed, cells were washed with PBS and fresh medium was added. For MTS assay, CellTiter96^®^ AQueous One Solution Reagent was used and absorbance at 490 nm was measured to calculate the % of metabolically active cells.

### 3.11. Confocal Microscopy Imaging

SKOV-3, MDA-MB-231 and HepG2 (2 × 10^5^ cells per well) cells were seeded into six-well plates with sterile glass coverslips (diameter 12 mm, Thermo Fischer Scientific (Waltham, MA, USA) and incubated overnight. After removing medium, cells were treated with 1.43 × 10^11^ particles/well Au@Pt-PEG-COOH (SKOV-3, MDA-MB-231 and HepG2); trastuzumab (73 μg/well; SKOV-3 and MDA-MB-231) or bioconjugate (1.43 × 10^11^ particles/well; SKOV-3 and MDA-MB-231) and incubated for 24 h. Further, protocol was analogous as reported previously [31]. Imaging with DAPI staining were performed with 408 and 488 nm, whereas for Hoechst 33258 staining 352 nm and 461 nm wavelengths were used. Images for Au@Pt-PEG-COOH and bioconjugate were carried out using bright field by transmitted light detector (T-PMT). All computer operations were accomplished with ZEN 2.1 software (Zeiss, Jena, Germany).

### 3.12. Isolation of Nuclei from HepG2 Cell Line

To evaluate whether Au@Pt conjugates are able to overcome nuclear envelope and go through the cytoplasm into the nucleus leading the cytotoxicity, isolation of nuclei was performed. For this purpose, Nuclei Isolation Kit: Nuclei EZ Prep (Sigma, St. Louis, MO, USA) was used and all of the preparation steps were realized according to the supplier protocol. Briefly, 5 × 10^6^ cells were seeded into ∅100 mm petri dish and incubated overnight. Next, medium was removed and 1.43 × 10^11^ Au@Pt-PEG-COOH particles per mL were suspended in growing medium. Cells were incubated for 24, 48 and 72 h at 37 °C in CO_2_ incubator. Then, medium was removed and cells were washed with cold PBS. Finally, Nuclei EZ Lysis Buffer was added, cells were lysed with the cell scraper and transformed into 15 mL centrifuge tube. After 5 min of incubation on ice, centrifugation at 4 °C for 5 min and washing with Lysis Buffer were performed. The last step was to disperse the pellet in Storage Buffer and prepare small-volume samples for ICP-MS platinum quantification. Additionally, yield of isolated nuclei was calculated by using hemocytometer and Trypan Blue staining.

## 4. Conclusions

Au@Pt-PEG-trastuzumab and Au@Pt-PEG-COOH conjugates were studied in terms of their application as radioactive analogues labeled with ^193m,195m^Pt. The performed studies have shown that Au@Pt-PEG-trastuzumab exhibits a high affinity to HER2 receptors, over 90% internalization and location in the perinuclear area. tested concentrations, cytotoxicity of the conjugate was not observed. However, when radioactive ^193m,195m^Pt emitting Auger electrons are applied, it is reasonable to expect a cytotoxic effect due to the perinuclear location and probable partial intranuclear location related to the radiation generation of ROS and RNS and their transport from the perinuclear area to the interior of the cell nucleus.

Unfortunately, in the studies related to HCC therapy, a suitable vector for targeting receptors on HepG2 cells was not found and was limited to testing of the Au@PtNP-PEG-COOH conjugate. However, despite the lack of a targeting molecule for inducing cytotoxicity of the Au@PtNP-PEG-COOH, a significantly lower concentration of compound was necessary to obtain a similar cytotoxicity effect than for platinum-coated nanoparticles and *cis*-platinum in other, comparable studies [12]. As was found, this cytotoxic effect is not related to the dissolution of Pt and the transport of Pt^2+^ cations into the cell nucleus. Despite this, it promises high toxicity when radioactive nanoparticles Au@^193m,195m^Pt are used because ROS and RNS will be generated near the nucleus by the Auger electrons. The obtained results are promising for further development of Au@^193m,195m^Pt nanoparticles conjugates as a novel Auger electron-emitting radiation nanomedicine for the treatment of HER2-positive breast and ovarian cancer and HCC therapy. Unfortunately, the intravenous injection of our radiobioconjugates is excluded, due to its relatively large size causing the accumulation of this compound in many organs, especially in the liver, lung and spleen as described in many papers where nanoparticle-based radiopharmaceuticals were used [32,33]. However, proposed radiobioconjugates can be successfully used in a new unconventional therapeutic method, nanobrachytherapy, where radioactive nanoparticles are intratumorally-injected directly into the tumor or cavity after tumor resection [34].

In presented part of our research, much attention has been paid to the chemical aspect of radiopharmaceutical design. As was mentioned, targeted Auger therapy is one of the most challenging in radiotherapy drug conceptualization due to very short cellular range and related to this necessarily of precise conjugate delivery into the cancerous cells. Therefore, our studies were also concentrated on biological evaluation of synthesized compounds including their impact on–physiologically and morphologically–different cancer cell lines. However, it must be emphasized that until ^193m,195m^Pt will be used, additional in vitro biological studies on three-dimensional cell cultures-spheroids (3D) as well as in vivo experiments must be performed. This research will be continued in near future and published in the next paper as the following part of this publication.

## Figures and Tables

**Table 1:** Hydrodynamic diameter and zeta potential of AuNPs and AuNPs covered by ~1 and ~6 layers of Pt atoms.

	Hydrodynamic Diameter [nm]	Zeta Potential [mV]
AuNPs	35.20 ± 0.52	−43.45 ± 0.64
Au@Pt (monolayer)	36.96 ± 0.60	−31.82 ± 0.16
Au@Pt (~6 layers)	39.02 ± 0.38	−29.85 ± 0.07

**Table 2:** Hydrodynamic size and zeta potential of synthesized bioconjugate.

	Hydrodynamic Diameter [nm]	Zeta Potential [mV]
Au@Pt	32.92 ± 0.16	−45.10 ± 1.27
Au@Pt-PEG-COOH	59.37 ± 1.05	−16.00 ± 0.85
Au@Pt-PEG-trastuzumab	88.86 ± 3.13	−30.95 ± 0.07
Au@Pt-PEG-trastuzumab (with PEG-COOH)	92.19 ± 5.20	−35.30 ± 0.71

**Table 3:** Platinum concentrations in the cytoplasm and cell nucleus. Platinum content converted to one cell.

	Cytoplasm [fg Pt/cell]	Pt/Au Ratio	Nuclei [fg Pt/nucleus]
**24 h**	1004 ± 33	1.23	<1
**48 h**	1067 ± 33	1.23	<1
**72 h**	1232 ± 12	1.20	<1

## Data Availability

The data presented in this study are available on request from the corresponding author.

## Sample Availability

Samples of the compounds are available from the authors.
